# Characterization of the Antigen-Specific CD4^+^ T Cell Response Induced by Prime-Boost Strategies with CAF01 and CpG Adjuvants Administered by the Intranasal and Subcutaneous Routes

**DOI:** 10.3389/fimmu.2015.00430

**Published:** 2015-08-28

**Authors:** Annalisa Ciabattini, Gennaro Prota, Dennis Christensen, Peter Andersen, Gianni Pozzi, Donata Medaglini

**Affiliations:** ^1^Laboratorio di Microbiologia Molecolare e Biotecnologia (LA.M.M.B.), Dipartimento di Biotecnologie Mediche, Università di Siena, Siena, Italy; ^2^Department for Infectious Disease Immunology, Statens Serum Institut, Copenhagen, Denmark

**Keywords:** CD4^+^ T cell priming, prime-boost strategies, MHC class II tetramers, CAF01, CpG, chemokines, lungs

## Abstract

The design of heterologous prime-boost vaccine combinations that optimally shape the immune response is of critical importance for the development of next generation vaccines. Here, we tested different prime-boost combinations using the tuberculosis vaccine antigen H56 with CAF01 or CpG ODN 1826 adjuvants, administered by the parenteral and nasal routes. Using peptide-MHC class II tetramers, antigen-specific CD4^+^ T cells were tracked following primary and booster immunizations. Both parenteral priming with H56 plus CAF01 and nasal priming with H56 plus CpG elicited significant expansion of CD4^+^ tetramer-positive T cells in the spleen; however, only parenterally primed cells responded to booster immunization. Subcutaneous (SC) priming with H56 and CAF01 followed by nasal boosting with H56 and CpG showed the greater expansion of CD4^+^ tetramer-positive T cells in the spleen and lungs compared to all the other homologous and heterologous prime-boost combinations. Nasal boosting exerted a recruitment of primed CD4^+^ T cells into lungs that was stronger in subcutaneously than nasally primed mice, in accordance with different chemokine receptor expression induced by primary immunization. These data demonstrate that SC priming is fundamental for eliciting CD4^+^ T cells that can be efficiently boosted by the nasal route and results in the recruitment of antigen-experienced cells into the lungs. Combination of different vaccine formulations and routes of delivery for priming and boosting is a strategic approach for improving and directing vaccine-induced immune responses.

## Introduction

Heterologous combinations of vaccine formulations for priming and boosting the immune system represent a strategic tool for the development of next generation vaccines. The combination of vaccines including vectors or adjuvants with different mechanisms of action for priming and boosting can contribute to the development of effective immune responses (1–6). In prime-boost vaccination strategies, different routes of delivery, such as mucosal and parenteral, can also be combined in order to induce immune responses in both the local and systemic compartments (7, 8).

A critical event in the initiation of the immune response to vaccination is the primary activation of vaccine antigen-specific T helper cells. T-cell priming indeed deeply influences both the magnitude and the quality of the immune response elicited by vaccination, as it is required for both the induction of high-affinity antibodies and the generation of long-lasting immune memory (9). Primary CD4^+^ T cell activation is therefore essential for orchestrating the subsequent adaptive immune response elicited by vaccination and its characterization in terms of magnitude, quality, and memory generation, is therefore of primary importance in vaccine design (9). CD4^+^ T-cell priming properties of different adjuvants (10–12), delivery systems (13, 14), and immunization routes (15–17), have been characterized for a rational design of effective prime-boost combinations (8). The route of immunization deeply influences the local and systemic immune response (18–20) and affects the polarization of CD4^+^ T effector cells into different helper subtypes. We have recently shown that the route used for priming but not for boosting, influences the Th1 or Th2 skewing, with a stronger Th1 polarization in mucosally primed mice (8). Additionally, we have shown that mice primed and/or boosted by the nasal route produce higher levels of IL-17A than mice primed-boosted systemically. Moreover, local effector responses are mainly dependent on mucosal boosting (8). Therefore, the use of combined prime-boost immunization can impact on the efficiency and the localization of the immune response to a vaccine formulation.

Here, we assessed the antigen-specific local and systemic CD4^+^ T cell response following parenteral–mucosal prime-boost combinations, with the *Mycobacterium tuberculosis* fusion protein H56 formulated with CAF01 or CpG ODN adjuvants. The fusion protein H56 is a promising tuberculosis (TB) vaccine candidate consisting of the antigens Ag85B fused to the 6-kDa early secretory antigenic target (ESAT-6) and the latency-associated protein Rv2660c (21), and it is a component of vaccine candidates currently in clinical trials (ClinicalTrials.gov Identifier numbers: NCT01967134, NCT01865487). Adjuvants deeply influence the quality of the adaptive immune response, and therefore, their selection is fundamental in a vaccine formulation. Two validated adjuvants already tested in clinical trials (22, 23) and with long pre-clinical track record were here selected for prime-boost combinations. CAF01 adjuvant is a liposomal adjuvant system composed of cationic liposome vesicles [dimethyldioctadecylammonium (DDA)] combined with a glycolipid immunomodulator component [trehalose 6,6-dibehenate (TDB); a synthetic variant of cord factor located in the mycobacterial cell wall] that act in synergy to enhance vaccine-specific immune responses (24). CAF01 has shown to promote vaccine depot formation, prolonging the release of antigens while targeting the antigens and immunomodulator to the same activated APCs (25). Pre-clinical studies with CAF01 have shown the induction of combined Th1 and Th17 responses and generation of robust, long-lived memory immunity (26, 27). We have recently characterized the induction of primary antigen-specific CD4^+^ T responses elicited by parenteral immunization with H56 and CAF01 confirming the differentiation of primed CD4^+^ T cells into Th1 and Th17 subtypes together with follicular T helper cells within draining lymph nodes (Prota et al., submitted). The other adjuvant selected, CpG ODN 1826 (CpG), is an agonist of the toll-like receptor 9 (TLR9) and belongs to the class of pathogens-associated molecular patterns (PAMPs) that interact with different receptors present on cells of the innate immune system (23). Several studies have shown that the co-administration of CpG ODN with vaccines administered by mucosal routes significantly increases local antigen-specific IgA and IgG levels systemically (23, 28, 29), and induces primary CD4^+^ T cells responses in the draining lymph nodes and in the spleens (10, 15). Clinical studies designed to evaluate CpG activity in humans have shown its safety and adjuvanticity (23).

In the present study, we have characterized the induction of antigen-specific CD4^+^ T-cell expansion and differentiation elicited by priming or prime-boost combinations of vaccine formulations, including H56 plus CAF01 or CpG adjuvants administered by intra nasal (IN) or subcutaneous (SC) routes. Antigen-specific T helper cells were identified employing MHC class II tetramers complexed with an Ag85B-derived peptide that includes an immunodominant epitope. Priming of Ag85B-specific CD4^+^ T cells was analyzed in the spleen and also in the lungs, the latter representing the main target organ for local immune response against *M. tuberculosis* infection. These studies provide an important contribution to the rational design of prime-boost combinations capable of eliciting local and systemic antigen-specific CD4^+^ T cells.

## Materials and Methods

### Mice

Eight-week-old female C57BL/6 mice, purchased from Charles River (Lecco, Italy) were maintained under specific pathogen-free conditions at the University of Siena and treated according to national guidelines (Decreto Legislativo 26/2014). All animal studies were approved by the Ethics Committee “Comitato Etico Locale dell’Azienda Ospedaliera Universitaria Senese” and the Italian Ministry of Health (number 4/2011, on date 20/07/2011).

### Immunizations

Subcutaneous immunizations were performed with the *M. tuberculosis* fusion protein H56 (22) (10 μg/mouse, Statens Serum Institut, Denmark) mixed with the adjuvant CAF01 (23) (250 μg DDA and 50 μg TDB/mouse, Statens Serum Institut), in a volume of 150 μl/mouse of TRIS HCl 10 μM. Nasal immunization was performed with H56 (10 μg/mouse) mixed with the adjuvant CpG ODN 1826 (20 μg/mouse, TCC ATG ACG TTC CTG ACG TT, Eurofins MWG Operon, Germany,) or with CAF01 (62.5 μg DDA and 12.5 μg TDB/mouse), in a volume of 20 μl/mouse of phosphate buffered saline solution (PBS, Sigma-Aldrich). For priming studies, mice were immunized at day 0 and sacrificed on day 7, while in prime-boost experiments, mice were immunized at day 0, boosted on week 4, and sacrificed 7 days later.

### Sample collection and cell preparation

Lungs and spleens were collected 7 days after primary or booster immunization. Spleens were mashed onto nylon screens (Sefar Italia, Italy) and washed two times in complete medium [RPMI medium (Lonza, Belgium), 100 U/ml penicillin/streptomycin, and 10% fetal bovine serum (GIBCO, USA)]. Lungs were collected as previously described (13), briefly after *in vivo* perfusion with PBS, organs were digested with collagenase D (1 mg/ml, Sigma-Aldrich) and DNase I (28 U/ml, Sigma-Aldrich) enzymes for 1 h at 37°C, mashed on nylon screens, and separated on lympholyte M cell separation density gradient centrifugation media (Cedarlane Laboratories, Canada).

### Flow cytometric analysis

Cells were seeded (5 × 10^6^ cells/well) in a V-bottom 96-well plate (SARSTED, USA) and incubated for 30 min at 4°C in Fc-blocking solution [complete medium plus 5 μg/ml of CD16/CD32 mAb (clone 93; ebioscience, CA, USA)]. PE-conjugated I-A(b) *M. tuberculosis* Ag85B precursor 280-294 (FQDAYNAAGGHNAVF), or PE-conjugated human class II-associated invariant chain peptide (PVSKMRMARPLLMQA) tetramers (NIH MHC Tetramer Core Facility, Emory University, Atlanta, GA, USA) were added at the concentration of 15 μg/ml. Samples were washed and stained on ice with PE-CY7-conjugated anti-CXCR3 (clone CXCR3-73), BV421-conjugated anti-CCR6 (clone 292L17, all purchased from Biolegend, CA, USA), HV500-conjugated anti-CD4 (clone RM4-5), and HV450-conjugated anti-CD44 (clone IM7, all purchased from BD biosciences, CA, USA). Samples were labeled with LIVE/DEAD Fixable Near IR Dead Cell Stain Kit according to the manufacturer instruction (Invitrogen, USA). Intracellular staining for BV605-conjugated anti-T-bet (clone 4b10, Biolegend, CA, USA) and PerCPCY5.5-conjugated anti-RORγt (clone Q31-378) (BD biosciences, CA, USA) was performed using the Foxp3 staining buffer set (eBioscience, CA, USA) according to the manufacturer instruction. Antibodies and tetramers were titrated for optimal dilution. About 10^6^ cells were stored for each sample acquired on LSR II flow cytometer (BD biosciences), and data were analyzed with FlowJo (TreeStar, OR, USA).

### Statistical analysis

One-way ANOVA and Tukey’s post test for multiple comparisons were employed to analyze frequencies of Ag85B-specific CD4^+^ T cells between groups receiving the same primary immunization and to analyze frequencies of Ag85B-specific CD4^+^ T cells detected in naïve mice and in groups only primed. Two-tailed Student’s *t*-test was employed to analyze statistical differences between the percentage of Ag85B-specific CD4^+^ T cells expressing transcription factors and chemokines receptors, in the two groups selected. Statistical significance was defined as *P* ≤ 0.05. Graphpad 4.0 software was used for analysis.

## Results

### Ag85B-specific CD4^+^ T cell response in the spleen following different prime-boost combinations

**Figure 1:**
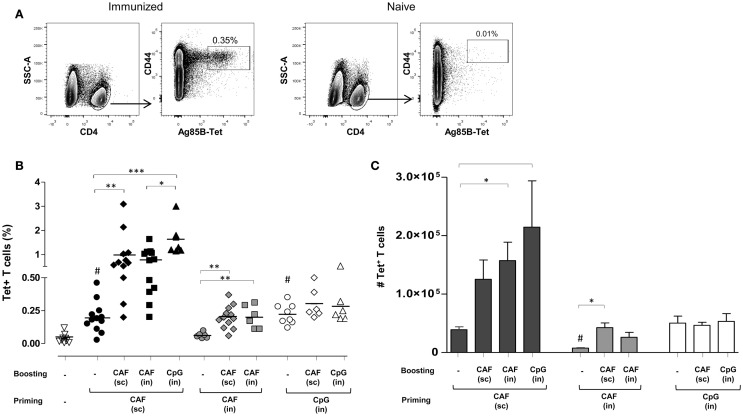
**Ag85B-specific CD4^+^ T cells in the spleen of mice immunized with different prime-boost combinations**. **(A)** Representative dot plots showing the identification of Ag85-specific T helper cells in immunized or naïve mice. Tet^+^ T cells were detected as CD44^high^ Tet-Ag85B^+^ cells gated on live CD4^+^ lymphocytes; percentages of Tet^+^ T cells respect to CD4^+^ T cells are reported. **(B)** Tet^+^ T cells in the spleen of mice primed (week 0) and boosted (week 4) with different vaccine combinations administered by parenteral or mucosal routes and assessed 7 days after the last immunization. The analysis shows the percentage of Tet^+^ T cells detected in the spleen of naïve mice (open inverted triangles), primed groups (black, gray and white circles), or prime-boost groups (diamonds, squares, and triangles). Vaccine combinations include parenteral priming with CAF01 followed by parenteral or nasal boosting with CAF01 or CpG (filled symbols), nasal priming with CAF01 followed by parenteral or nasal boosting with CAF01 (gray symbols), or nasal priming with CpG followed by parenteral or nasal boosting with CAF01 or CpG (open symbols). Statistical analysis was performed using one-way ANOVA and Tukey’s post test for multiple comparisons among groups receiving the same primary immunization (*P ≤ 0.05, **P ≤ 0.01, and ***P ≤ 0.001) as well as among mice receiving only the primary immunization versus *naïve* animals (^#^*P* ≤ 0.05). **(C)** Absolute number of Tet^+^ T cells in the spleen, elicited by priming or prime-boost combinations, reported as mean ± SEM of 6–12 animals per group from two independent experiments. Statistical analysis was performed using one-way ANOVA and Tukey’s post test for multiple comparisons among groups receiving the same primary immunization (*P ≤ 0.05) as well as among mice receiving only the primary immunization (^#^*P* ≤ 0.05 versus SC priming with CAF01 and IN priming with CpG).

Antigen-specific T helper responses were evaluated following heterologous prime-boost immunizations obtained with the two adjuvants CAF01 and CpG and parenteral/mucosal delivery routes. CD4^+^ T cells specific for the immunodominant epitope of Ag85, that is part of the H56 fusion protein, were identified using Ag85B_280–294_-complexed MHC class II tetramers. Tetramer-binding CD4^+^ (Tet^+^) T cells were assessed in spleens 7 days after primary or secondary immunizations. Representative dot plots showing Tet^+^ T cells frequency in immunized or naïve animals are shown in Figure 1A. Staining specificity was determined using a control tetramer complexed with an unrelated antigen that showed a level of staining below 0.02% (data not shown).

In Figure 1B, frequencies of Tet^+^ T cells detected in naïve mice (inverted triangles) or elicited by priming (circles) or prime-boost combination (diamonds, squares, or triangles) are reported. Priming with both CAF01 by the SC route (black circles) and CpG by the IN route (open circles) induced a significant increase of Tet^+^ T cell frequency compared to naïve animals (open inverted triangles, *P* ≤ 0.001), while CAF01 administered by the IN route was not effective in stimulating antigen-specific T-cell priming (gray circles).

Mice SC primed with H56 and CAF01 generated stronger peptide-specific CD4^+^ T cell responses following booster immunizations compared to nasally primed mice. Indeed, primed cells were efficiently boosted by both SC immunization with CAF01 (black diamonds) and IN administration of CpG (black triangles) with a significant increase of T-cell responses compared to the group parenterally primed with CAF01 (*P* ≤ 0.01 and *P* ≤ 0.001, respectively). IN boosting with CAF01 improved the frequency of Tet^+^ T cells (black squares), but the increase was not statistically significant compared to primed mice using the analysis of variance test.

Animals primed by the IN route with the two adjuvants CAF01 and CpG were low responders to booster immunization compared to parenterally primed mice. Indeed, although the frequency of Tet^+^ T cells induced by IN administration of CpG (open circles) was similar to the one observed upon SC priming with CAF01 (black circles) these T cells could not be boosted, as shown by the very low frequencies of Tet^+^ T cells upon booster immunizations (Figure 1B). The expansion detected in nasally primed mice upon SC boosting with CAF01 (gray and white diamonds) resembled the primary response elicited by parenteral priming with CAF01 (black circles), suggesting that the observed response could merely be a result of the parenteral immunization.

Since the relative frequency of Tet^+^ respect to total CD4^+^ T cells can be affected by the enhancement of total CD4^+^ T cells upon boosting, we also analyzed the absolute number of Ag85B-specific CD4^+^ T cells within the spleen. The higher increase of Tet^+^ T cell number in the spleens upon booster immunization was observed in mice that had been parenterally primed with CAF01 and nasally boosted, but not in groups nasally primed (Figure 1C).

### Ag85B-specific CD4^+^ T cell response in lungs following different vaccine combinations

**Figure 2:**
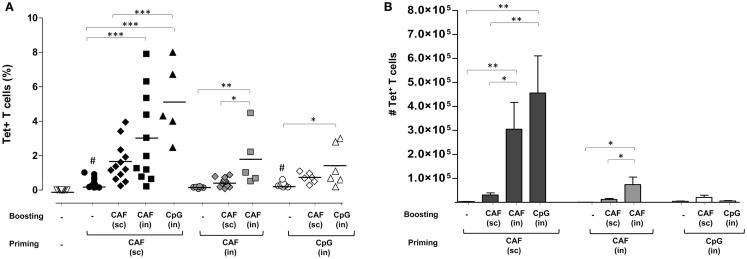
**Ag85B-specific CD4^+^ T cells in the lungs of mice immunized with different prime-boost formulations**. **(A)** Tet^+^ T cells in the lungs of mice primed (week 0) and boosted (week 4) with different vaccine combinations administered by parenteral or mucosal routes and assessed 7 days after the last immunization. The analysis shows the percentage of Tet^+^ T cells detected in lungs of naïve mice (open inverted triangles), primed groups (black, gray, and white circles), or prime-boost groups (diamonds, squares, and triangles). Vaccine combinations included parenteral priming with CAF01 followed by parenteral or nasal boosting with CAF01 or CpG (filled symbols), or nasal priming with CAF01 followed by parenteral or nasal boosting with CAF01 (gray symbols), or nasal priming with CpG followed by parenteral or nasal boosting with CAF01 or CpG (open symbols). Statistical analysis was performed using One-way ANOVA and Tukey’s post test for multiple comparisons among groups receiving the same primary immunization (*P ≤ 0.05, **P ≤ 0.01, and ***P ≤ 0.001) as well as among mice receiving only the primary immunization versus *naïve* animals (^#^*P* ≤ 0.05). **(B)** Absolute number of Tet^+^ T cells in lungs elicited by priming or prime-boost combinations reported as mean ± SEM of 6–12 animals per group from 2 to 3 independent experiments.

Expansion of Ag85B-specific CD4^+^ T cells was analyzed in lungs, a target effector site for vaccines aimed at fighting airway pathogens. Primary immunization by SC route with CAF01 (black circles, 0.4%) and IN route with CpG (open circles, 0.3%), but not CAF01 (gray circles) induced a significant increase of Tet^+^ T cell frequency compared to naïve animals (open triangles, *P* ≤ 0.001 and *P* ≤ 0.05, respectively). As observed in the spleen, parenterally primed-mucosally boosted mice showed the stronger Tet^+^ T cell response compared to the other combinations, highlighting the recruitment effect exerted by the nasal route employed for boosting (Figure 2A). The highest percentage of Tet^+^ T cells was observed in mice parenterally primed with CAF01 and nasally boosted with CpG (black triangles) that was about 5% of CD4^+^ T cells of the lungs, while about 3% were detected upon IN boosting with CAF01 (black, squares). An increase in the frequency of Tet^+^ T cells was observed also in the group subcutaneously boosted with CAF (black diamonds), although not statistically significant (Figure 2A).

Cells primed by the IN route with both CAF01 and CpG were much less prone to expand upon nasal boosting compared to parenterally primed cells (Figure 2A). This was clearly shown by the absolute number of antigen-specific CD4^+^ T cells found in the lungs that highlights the strong increase in parenterally primed-nasally boosted mice (both with CAF01 and CpG) compared to the other prime-boost combinations (Figure 2B). The stronger recruitment of tetramer-specific CD4^+^ T cells into lungs upon IN boosting correlated with a high afflux of total lymphocytes into the organs (data not shown). This analysis shows not only the recruitment effect exerted by IN boosting but also the fundamental role of primary immunization in generating cells capable of reactivating upon secondary immunization.

In conclusion, parenteral priming with CAF01 adjuvant followed by nasal boosting with CpG was the prime-boost combination that elicited the stronger peptide-specific CD4^+^ T cell response in both spleen and lungs.

### Phenotypic characterization of Tet^+^ T cells elicited by nasal or parenteral priming and boosted by IN route

**Figure 3:**
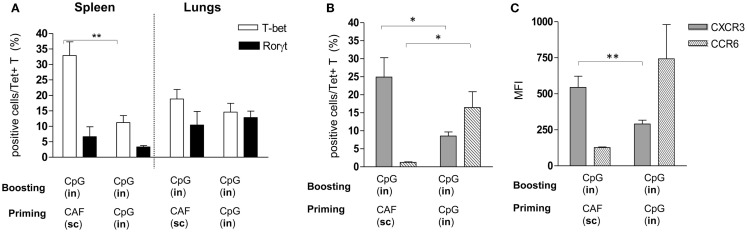
**Phenotypic analysis of Tet^+^ T cells detected in spleen and lungs**. Tet^+^ T cells detected in spleens and lungs of mice primed by SC route with CAF01 or by IN route with CpG and nasally boosted with CpG were characterized for the expression of transcription factors **(A)** and CXCR3 and CCR6 chemokine receptors **(B,C)**. **(A)** Percentage of RORγt (filled bars) and T-bet (empty bars) positive cells among tetramer-binding CD4^+^ T cells detected in mice immunized with the vaccine combinations are reported in *x* axis. **(B,C)** Percentage **(B)** and mean fluorescent intensity [MFI, **(C)**] of CXCR3-positive (gray bars) and CCR6-positive cells (striped bars) among Tet^+^ T cells assessed in lungs of mice immunized with the vaccine combinations are reported in *x* axis. Bars represent the mean ± SEM of six mice per group and it is representative of two independent experiments. Statistical analysis was performed using the two-tailed Student’s *t*-test. *P < 0.05, **P < 0.01.

The analysis of Tet^+^ T cells in the lungs highlighted the important role of the IN route for boosting, and the different recruitment effect exerted on cells that had been primed by parenteral or nasal immunizations. We therefore investigated if Tet^+^ T cells, elicited by IN immunization with CpG or SC immunization with CAF01 and then boosted with CpG by the nasal route, showed differences in terms of subtype differentiation and chemokine receptor expression. Both adjuvants are known to stimulate Th1 subtype differentiation (26, 30) while Th17 had been reported with CAF01 (27), we therefore analyzed the intracellular expression of T-bet and RORγt, the respective master transcription factors (31, 32) among Tet^+^ T cells. Frequency of T-bet-positive cells was significantly higher in the spleen of parenterally primed-nasally boosted mice compared to mucosally primed-boosted animals, while in the lungs, both T-bet and RORγt positive cells were observed without significant differences between the two vaccination strategies (Figure 3A). We therefore assessed if Tet^+^ T cells recruited into lungs in parenterally primed-nasally boosted mice expressed different chemokine receptors with respect to the nasally primed-boosted group. In the first group, there was a higher percentage of Tet^+^ T cells that expressed CXCR3 (Figure 3B) with a significant higher intensity (Figure 3C) compared to the mucosally primed-boosted group, which in turn showed a higher expression of CCR6 (Figure 3B). The expression of CXCR3 or CCR6 in lung cells was not dependent on the T cell subtype, since no significant differences were observed among T-bet^+^ and RORγt^+^ subgroups (data not shown).

In conclusion, the different recruitment of peptide-specific CD4^+^ T cells into lungs elicited by the two immunization strategies could be due to the different chemokine receptors expressed by T cells upon primary activation.

## Discussion

The induction of primary and secondary peptide-specific CD4^+^ T cell responses was assessed and compared following different prime-boost immunization strategies based on the combinations of different vaccine formulations and routes of delivery. Our data show that parenteral priming with H56 antigen and CAF01 adjuvant followed by nasal boosting with vaccine antigen and CpG is the prime-boost combination able to elicit the strongest peptide-specific CD4^+^ T cell response in both spleen and lungs, compared to the other homologous or heterologous prime-boost combinations tested.

Parenteral priming with CAF01 adjuvant elicited the highest number of antigen-specific CD4^+^ T cells capable of responding to booster immunization, whereas the T cell response detected after nasal priming was poorly responsive to recall immunization. While nasal priming with CAF01 did not induce proliferation of Tet^+^ T cells, suggesting that CAF01 is not optimal for CD4^+^ T cell priming by the IN route, nasal priming with the CpG adjuvant elicited a significant expansion of Ag85B-specific CD4^+^ T cells that, in terms of absolute numbers and frequency of primed cells, was comparable to that observed in parenterally primed mice. Nevertheless, these primed CD4^+^ T cells did not respond to both homologous and heterologous secondary immunization, suggesting that memory cells were not efficiently elicited by the IN priming. The low capacity of nasal priming to generate booster responder CD4^+^ T cells was noted with both CAF01 and CpG adjuvants that differ in structure and mechanism of action, being a liposomal adjuvant system incorporating the C-type lectin Mincle agonist TDB and a pure TLR9 ligand, respectively. This observation demonstrates that different conditions of primary immunization differently affect the immunological memory process, an event shaped by many factors that still needs to be understood in its complexity. A critical factor may be the timing of antigen presentation; indeed, it has been recently shown that effector CD4^+^ T cells need a late contact with antigen presenting cells 4–6 days after immunization to survive to the contraction phase and generate a pool of memory CD4^+^ T cells (33). We previously characterized within draining lymph nodes the antigen presentation of a fluorescent model antigen administered with CpG by the IN route, showing that the presentation peaked 12 h after immunization and was completely exhausted within 3 days (15). This is different from parenteral immunization with a vaccine antigen and CAF01, where the duration of antigen presentation was at least 5 days (25). However, other mechanisms, such as local innate immune responses, costimulatory and cytokine signals during the antigen presentation event, different recirculation of primed T cells, can differently influence the immunological memory process upon systemic and mucosal priming.

IN immunization with both CAF01 and CpG adjuvants was able to stimulate a recall response to parenterally primed mice. Indeed, nasal boosting was essential for the recruitment in the lungs of activated CD4^+^ T cells. The optimal prime-boost combination for eliciting activated CD4^+^ T cells, not only in the spleen but also in the lungs, appeared to be parenteral priming with CAF01 followed by nasal boosting with CpG. The dissemination of Ag-specific CD4^+^ T cells in the lungs elicited by nasal booster immunization was largely dependent on the T-cell priming event. Indeed, parenterally primed-nasally boosted mice exhibited more than a 100-fold increase in the amount of Ag85B-specific CD4^+^ T cells in the lungs upon secondary immunization, while the recruitment of specific CD4 T cells after mucosal boosting was extremely low in nasally primed mice. This behavior was not due to a different subpopulation profile generated during the priming event by CAF01 or CpG, since primed CD4^+^ T cells differentiated into similar T helper subtypes that expressed T-bet or RORγt, without significant differences between the two vaccine formulations. On the contrary, different chemokine receptors were induced on the surface of Tet^+^ T cells primed by parenteral or nasal routes. CXCR3, known to be involved in lung trafficking of T cells (34), was expressed more highly on parenterally than mucosally primed Tet^+^ T cells, which in turn expressed more CCR6, a marker associated with Th17 and IL-17 secretion (35). Therefore, the pattern of chemokine receptors appears to be differently regulated on cells primed by SC or IN route, and this can influence the cell migration. These data are particularly relevant when vaccines able to induce local immune responses are required. Indeed, the generation of pulmonary antigen-specific CD4^+^ T cells is of key importance for the design of TB vaccines aimed at inducing local immune responses in the respiratory tract (36, 37). Of course, the recruitment of immune cells into lungs needs to be tightly regulated to effectively favor protective mechanisms while avoiding or minimizing immunopathology (37). This is a crucial aspect in the field of TB vaccine development, which has been complicated and delayed by major gaps in the understanding of how immunity to TB manifests itself at the site of infection in the lung (38).

Despite many potential advantages of mucosal administration of vaccines, today only a few vaccines are licensed for nasal or oral administration (39–41). Heterologous combinations of administration routes offer the possibility of generating local immune response, using the mucosal route for boosting cells previously primed by the parenteral route.

In the current study, no severe adverse effects indicative of immunopathology in the respiratory tract of parenterally primed and nasally boosted mice were observed, beyond an increase in total T cell numbers in the lung. However, more detailed examination of airway responsiveness in future models would be required to absolutely determine the safety of this prime-boost approach.

In conclusion, our data show the induction of an efficient CD4^+^ T cell priming upon SC, but not IN administration of CAF01 with generation of cells that can be efficiently boosted by both parenteral and nasal routes. On the contrary, IN priming with both CAF01 and CpG adjuvants did not elicit cells capable of reactivation upon booster immunization. The stronger CD4^+^ T cell activation in both spleen and lungs was induced by parenteral priming with H56 and CAF01 followed by nasal boosting with vaccine antigen and CpG, compared to the other homologous or heterologous prime-boost combinations tested. These data demonstrate that parenteral priming followed by nasal boosting induces CD4^+^ T cell responses in lungs and spleen. Priming and booster immunization are therefore strategic events in the induction of vaccine immune responses, and the choice of adjuvants or vectors together with the route of immunization plays a fundamental role for improving vaccine immunogenicity and directing the recruitment of antigen-specific cells into specific effector sites, such as the lungs.

## Conflict of Interest Statement

The authors declare that the research was conducted in the absence of any commercial or financial relationships that could be construed as a potential conflict of interest.
